# Elbow-joint morphology in the North American ‘cheetah-like’ cat *Miracinonyx trumani*

**DOI:** 10.1098/rsbl.2022.0483

**Published:** 2023-01-25

**Authors:** Borja Figueirido, Alejandro Pérez-Ramos, Anthony Hotchner, David Lovelace, Francisco J. Pastor, Alberto Martín-Serra

**Affiliations:** ^1^ Departamento de Ecología y Geología, Facultad de Ciencias, Universidad de Málaga, Málaga 29071 Spain; ^2^ Department of Geoscience, University of Wisconsin-Madison, Madison, WI 53706, USA; ^3^ Departamento de Anatomía y Radiología, Museo de Anatomía, Universidad de Valladolid, Valladolid 47005, Spain

**Keywords:** *Miracinonyx trumani*, predatory behaviour, elbow joint, convergence, pronghorn

## Abstract

The North American cheetah-like cat *Miracinonyx trumani* is an extinct species that roamed the Pleistocene prairies 13 000 years ago. Although *M. trumani* is more closely related to the cougar (*Puma concolor*) than to the living cheetah (*Acinonyx jubatus*), it is believed that both *A. jubatus* and *M. trumani* possess a highly specialized skeleton for fast-running, including limbs adapted for speed at the expense of restricting the ability of prey grappling. However, forelimb dexterity of *M. trumani* has not been yet investigated. Here, we quantify the 3D-shape of the humerus distal epiphysis as a proxy for elbow-joint morphology in a sample of living cats to determine whether the extinct *M. trumani* was specialized to kill open-country prey using predatory behaviour based on fast running across the prairies and steppe terrains of the North American Pleistocene. We show that *M. trumani* had an elbow morphology intermediate to that of *P. concolor* and *A. jubatus*, suggesting that *M. trumani* had a less specialized pursuit predatory behaviour than *A. jubatus*. We propose that *M. trumani* probably deployed a unique predatory behaviour without modern analogues. Our results bring into question the degree of ecomorphological convergence between *M. trumani* and its Old World vicar *A. jubatus*.

## 1. Introduction

Among land vertebrates, the pronghorn ‘antelope’ (*Antilocapra americana*; Antilocapridae; Artiodactyla) is capable of a top speed of 100 km h^−1^ and second only to the cheetah (*Acinonyx jubatus*) [1]. The excessive speed of the pronghorn has been explained as an evolutionary response to predation from the now-extinct ‘cheetah-like’ cat *Miracinonyx trumani* [2], a formidable predator that roamed North America's Pleistocene steppes and prairies 13 000 years ago [3]. *Miracinonyx trumani* is related to *Puma concolor* [4,5] but a remarkable morphological similarity with *A. jubatus* has been documented [5,6]*,* presumably as a result of evolutionary convergence towards a fast-pursuit predatory behaviour [4]. Although the co-evolution between the modern pronghorn and the extinct American ‘cheetah’ has been used as a textbook example of evolutionary anachronism [7], recent studies suggest that *M. trumani* was a generalist predator [8] that retained the ability of prey grappling [9,10]. However, cursorial locomotion and forearm manipulation are conflicting functions [11–16] because pursuit predators possess limbs adapted for speed and locomotor efficiency at the expense of restricting the joint motion to the parasagittal plane, which limits prey grappling [11–16]. An established morphological indicator of forearm manoeuvrability and, hence, of predatory behaviour can be found in the articular surface of the humeral distal epiphysis or elbow joint [11–16].

Here, we use 3D geometric morphometrics to explore elbow-joint morphology in living carnivores of known predatory behaviour, including the Old World *A. jubatus* and *P. concolor*. The intent is to ascertain the likely predatory mode of the extinct *M. trumani* and to consider how this may impact hypotheses relating to adaptive convergence between true and putative cheetahs. Specifically, we test whether the North American *M. trumani* possessed a limited capability for prey grappling comparable to *A. jubatus*, as would be expected for a potential predator of fast prey across the prairies and steppe terrains of North America during the Pleistocene.

## 2. Material and methods

The distal ends of the humerus of 26 specimens belonging to 11 felid species (ten living and one extinct, *M. trumani*) were scanned with either surface scanning or micro-CT scanning (table 1, electronic supplementary material). The humerus of *M. trumani* (KUVP-51277) was unearthed from Natural Trap Cave (northern Wyoming, USA) with an age of *ca* 23–25 ka [17]. We used the distal end of the humerus because although the elbow articulation is also composed of the proximal ends of radius and ulna, this morphological structure is a well-established proxy for elbow-joint morphology (e.g. [11–16]).

**Table 1.** List of specimens included in this study. The scanning method is indicated as follows: (1) surface scanning using a EinScan Pro 2X Plus surface scanner; (2) surface scanning using an Artec Spider or an Artec EVA (Artec Corp., Luxembourg); (3) micro-CT-scanning at the Wisconsin Institute for Medical Research's Imaging Services Department of the University of Wisconsin with a GE Medical System Discovery model CT750 in Helicoidal mode and at Vithas Center (Málaga) using a GE Medical systems (Brivo CT385 Series); (4) the IMNH 996 and UF 25908 were downloaded from MorphoSource (https://www.morphosource.org/). The specimen IMNH 996 (ark:/87602/m4/M104608) was scanned with a Faro Edge Arm at the Idaho Virtualization Lab of Idaho State University and the specimen UF 25908 was micro-CT scanned at Florida Museum of Natural History (ark:/87602/m4/M31935). MNHN, Muséum National d'Histoire Naturelle (Paris, France); AMNH, American Museum of Natural History (New York, USA); MAV, Museo Anatómico de Valladolid (Valladolid, Spain); FMNH, Field Museum of Natural History (Chicago, USA); NMS, National Museum of Scotland (Edinburgh, UK); KUVP, University of Kansas Vertebrate Paleontology collections (Lawrence, USA); NHMUK, Natural History Museum (London, UK); IMNH, Idaho Museum of Natural History (Pocatello, USA); UWZS: University of Wisconsin Zoological Collection; UF, Florida Museum of Natural History, University of Florida (Gainesville, USA).

species	ID	museum	method
*Acinonyx jubatus*	1863-24	MNHN	1
*Acinonyx jubatus*	890-14	MNHN	1
*Acinonyx jubatus*	1901-541	MNHN	1
*Acinonyx jubatus*	1907-596	MNHN	1
*Acinonyx jubatus*	1932-442	MNHN	1
*Acinonyx jubatus*	1933-124	MNHN	1
*Acinonyx jubatus*	1996-287	MNHN	1
*Acinonyx jubatus*	1998-1942	MNHN	1
*Acinonyx jubatus*	1998-1981	MNHN	1
*Acinonyx jubatus*	2009-244	MNHN	1
*Acinonyx jubatus*	119657	AMNH	2
*Acinonyx jubatus*	6075	MAV	3
*Caracal caracal*	113794	AMNH	2
*Leptailurus serval*	44438	FMNH	2
*Leopardus wiedii*	2016.12.1	NMS	2
*Miracinonyx trumani*	54342	KUVP	3
*Neofelis nebulosa*	104730	FMNH	2
*Panthera leo*	85144	AMNH	2
*Panthera onca*	35571	AMNH	2
*Prionailurus viverrinus*	1860.7.22.22	NHMUK	2
*Puma concolor*	996	IMNH	4
*Puma concolor*	MAV 3087	MAV	3
*Puma concolor*	MAV 409	MAV	3
*Puma concolor*	32281	UWZS	3
*Puma concolor*	25908	UF	4
*Uncia uncia*	119662	AMNH	2

The meshes obtained from surface scanners were processed using the software associated with the scanners, ExScan Studio Pro (Shinning 3D) or Artec Studio 12 (Artec Corp., Luxembourg), and exported in .ply. These meshes were repaired with the software Geomagic Essentials (3D System, NC, USA). The CT-scanned specimens were processed using 3D Slicer [18].

**Figure 1.**
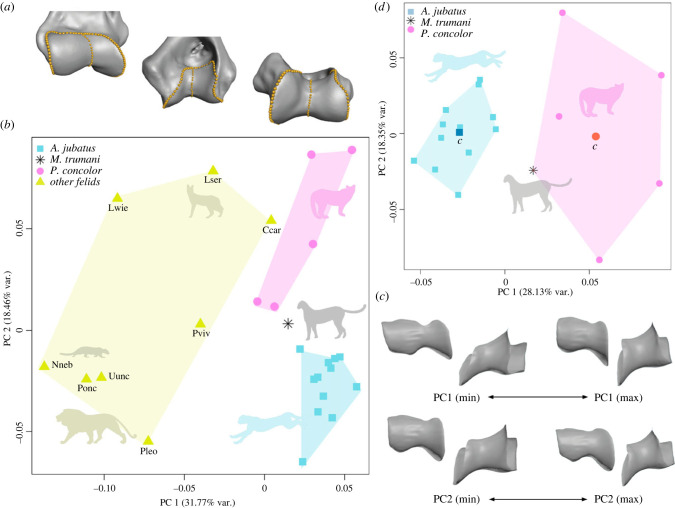
Analysis of the elbow joint in *M. trumani* and other felids. (*a*) Landmarks digitized to capture the three-dimensional shape of the elbow joint. The elbow of *M. trumani* (mirrored) as an example. From top to bottom: anterior, posterior and inferior (distal) views. (*b*) Bivariate graph depicted from the first two eigenvectors obtained from PCA; (*c*) shape changes accounted for by the first two eigenvectors. (*d*) Bivariate graph depicted from the first two eigenvectors obtained from PCA for the restricted sample of *M. trumani*, *P. concolor* and *A. jubatus*, where ‘*c*’ is the centroid. The convex hulls are defined according to the results obtained from *K*-means analysis—i.e. *K*-means analysis classified *M. trumani* with *Puma* and not with *Acinonyx*. Ccar, *Caracal caracal*; Lser, *Leptailurus serval*; Lwie, *Leopardus wideii*; Nneb, *Neofelis nebulosa*; Pleo, *Panthera leo*; Ponc, *Panthera onca*; Pviv, *Prionailurus viverrinus.* Silhouettes are not to scale.

One of us (AM-S) digitized two semilandmark rows to characterize the main morphological features of the distal articular surface. The first row (no. 1) follows the boundary of the articular surface surrounding the trochlea and capitulum (figure 1*a*), and the second row (no. 2) runs through the groove that separates these structures (figure 1*a*). The digitization was carried out using the software Avizo v.9 (Fisher Scientific). The raw 3D coordinates were uploaded into R environment [19] and processed to: (i) mirror the semilandmark configurations obtained from left humeri to have all of them as right humeri; (ii) standardize the number of semilandmarks of each row (119 for no. 1 and 27 for no. 2) and (iii) rearrange the semilandmarks at equal distances between them. These new semilandmark coordinates were incorporated into the analyses. First, we performed a Procrustes superimposition [20] sliding semilandmarks using minimum bending energy criterion [21] with function *gpagen* from Geomorph package [22]. Next, we carried out a principal components analysis (PCA) and generated the shape deformations of the first two principal components (PCs) using warping procedures over a 3D mesh of the distal articular surface in Geomorph [22].

Subsequently, we selected only the specimens of *A. jubatus*, *P. concolor* and *M. trumani* to focus on the morphological differences between these two living species and to assess for similarities between the elbow topology of *M. trumani* and either *A. jubatus* or *P. concolor*. Then, we repeated the Procrustes superimposition and PCA described above with this subsample, and we computed a *K*-means [23] analysis with two groups (*K* = 2) to classify *M. trumani* within one of these two living species because other classification methods, such as linear discriminant analyses, should be avoided when the number of variables is very high [24].

We also generated the average shape of the two resulting groups and we compared them with the shape of the distal articular surface of *M. trumani*. Topological deviations between these meshes were measured using Geomagic essentials software (Raindrop Corporation, Morrisville, NC, USA). We performed a morphing procedure using the surface model of the elbow joint of the specimen *P. concolor* (no. 409) as a starting shape to the average shape of *P. concolor*, *A. jubatus* and to the elbow of *M. trumani*. As the three models start from the same standard model (i.e. same number of mesh triangles and surface areas), the differences obtained in the topological deviation analysis will only be attributable to those shape changes applied to the three models.

## 3. Results

Figure 1*b* shows the bivariate plot depicted from the first two eigenvectors (PCs) obtained from a PCA of elbow shape. The first PC mainly separates out the *Puma*–*Acinonyx* lineage (figure 1*b*) according to their more squared and shorter (in medio-lateral direction) articular surface, as well as their shallow trochlear groove (figure 1*c*). By contrast, the second PC mainly separates *A. jubatus* from *P. concolor* (figure 1*c*), as the former has a more proximally expanded capitulum and a more vertical trochlear crest (figure 1*c*). The elbow of *M. trumani* clustered between the range of elbow shape variation of *P. concolor* and *A. jubatus* (figure 1*b*) but it was classified with *P. concolor* by *K*-means analysis (total sum of squares, 0.1196; between-clusters sum of squares, 0.0283; percentage of variance explained by the two groups, 23.67%; figure 1*d*).

**Figure 2.**
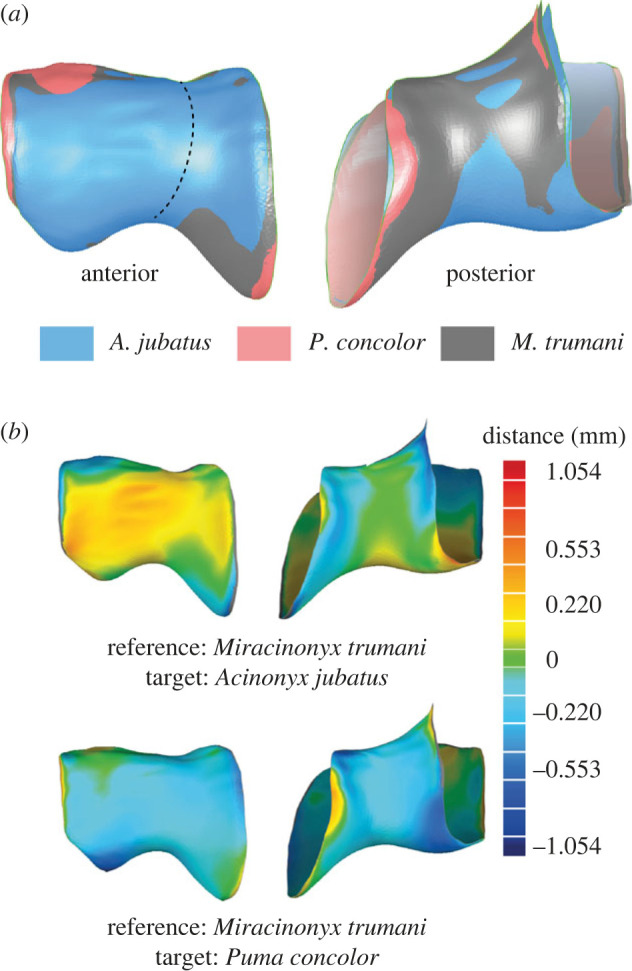
Elbow joint topological deviations in *M. trumani*, *A. jubatus* and *P. concolor* illustrated with the distal end of the right humerus. (*a*) Topological superimposition analysis of the three models showing differences between their elbows. (*b*) Topological deviations analysis between the elbows of *M. trumani* (reference) compared to that of *A. jubatus* and *P. concolor* (targets) separately. Warm colours are positive deviations of the target elbow and cold colours are negative deviations of the target. Green colours represent similar topologies between the target and the reference model. Distance units in mm.

Our topological analysis among the elbows of *P. concolor*, *A. jubatus* and *M. trumani* (figure 2*a*) shows that the trochlea crest of *P. concolor* is projected distally to a greater extent than *A. jubatus.* The capitulum of *A. jubatus* is highly curved, while *P. concolor* has the least curvature in this region. Both the trochlea crest and the capitulum curvature of *M. trumani* are somewhat intermediate of those of *P. concolor* and *A. jubatus*, but they are more similar to that of the former*.*

The quantifications of the topological deviations between *M. trumani* and both living taxa are shown separately in figure 2*b*. The capitulum and trochlea groove are more expanded in *A. jubatus* than in *M. trumani*, but the trochlea crest is less expanded in *A. jubatus*. The most distal part of the capitulum of *A. jubatus* is similar to that of *M. trumani*. The trochlea groove of the two models is almost identical. The elbow of *P. concolor* is much more similar to that of *M. trumani*, but with negative values close to zero in their topological deviations. Both the trochlea crest and the most lateral part of the capitulum at the proximal edge are similar in both taxa. The most distal part of the capitulum of *P. concolor* exhibits negative values relative to the topology of *M. trumani*, suggesting that *P. concolor* possesses a capitulum crest much flatter than *M. trumani*.

## 4. Discussion

Extreme pursuit predators such as *A. jubatus* possess limbs adapted for speed at the expense of restricting the joint motion to the parasagittal plane. This restriction entails the loss of the ability to supinate the forearm, which is essential for prey grappling [11–16]. As shown in figure 1*a*, *M. trumani* plots between the range of elbow shape variation of its closest living relative (*P. concolor*) and its Old World counterpart (*A. jubatus*), suggesting that there is no modern analogue for the elbow morphology of *M. trumani*.

The *K*-means analysis indicates that the elbow of *M. trumani* clusters with that of *P. concolor* and not with that of *A. jubatus*. This positioning of the *M. trumani* elbow from Natural Trap Cave does not necessarily reflect a specialized *Puma*-like predatory behaviour. Rather, it shows the retention of the ability to supinate the forelimb to grapple prey, unlike the condition seen in *A. jubatus* [11–16]. The superior ability of *M. trumani* to supinate the forelimb compared to that of *A. jubatus* also explains the retention of fully retractable claws, unlike *A. jubatus*, which are essential to immobilize relatively large prey prior to delivering a killing bite [25]. On the other hand, *M. trumani* exhibited a brachial index (a proxy for the degree of cursoriality) that is closer to that of *A. jubatus* than to *P. concolor* (table 2). Moreover, the higher brachial index of *M. trumani* relative to other ambush felids, such as the leopard (*Panthera pardus*), the jaguar (*Panthera onca*) or the tiger (*Panthera tigris*), suggests that *M. trumani* was a more cursorial form, but it retained fully retractable claws and some degree of supination at the elbow, which reinforces the idea that there is no modern analogue for the predatory behaviour exhibited by *M. trumani.* However, it is worth noting that the brachial index for the living lion (*Panthera leo*), which is an ambush predator that inhabits open habitats [15], is very similar to that of *M. trumani* (table 2).

**Table 2.** Values of brachial index for *M. trumani*, *P. concolor* and *A. jubatus*, as well as other ambush felids. The brachial index of *M. trumani* was calculated as radius length/humerus length × 100 using the values published by Van Valkenburgh *et al.* [5].

species	brachial index	reference(s)
*A. jubatus*	102.8, 103.3	[5,26]
*P. concolor*	83.8, 89.5	[5,21]
*M. trumani*	98.3	[5]
*P.onca*	86.8	[21]
*P. pardus*	90.5	[21]
*P. tigris*	89.8	[21]
*P. leo*	98.3	[21]

We propose that *M. trumani* was not as specialized as *A. jubatus* for deploying a predatory behaviour based on fast running and it probably deployed a predatory behaviour without modern analogues. We also bring into question the degree of ecological convergence between the Old World cheetah and the extinct North American ‘cheetah-like’ cat, as recently proposed by previous authors (e.g. [27]).

Therefore, although our results are focused on a single specimen of *M. trumani*, they cast doubts on the ‘anachronist’ hypothesis proposed to explain the excessive speeds of modern pronghorns as a result of a coevolutionary relationship with the extinct *M. trumani*. On the other hand, the excessive speeds of the modern pronghorn could be explained as an evolutionary by-product of its adaptation for decreasing transport costs in response to the spread of grassy habitats [28].

## Data accessibility

The data are provided in the electronic supplementary material [29], which comprises the raw landmark coordinates of those landmarks digitized on the distal end of the humerus of 26 specimens belonging to 11 felid species (10 living and one extinct, M. trumani) scanned with either surface scanning or micro-CT scanning, and the scanning protocol followed. The meshes obtained from surface scanners were processed using the software associated with the scanner, ExScan Studio Pro (Shinning 3D) or Artec Studio 12 (Artec Corp., Luxembourg), and exported in .ply. These meshes were repaired with the software Geomagic Essentials (3D System, NC, USA). The CT-scanned specimens were processed using 3D Slicer.
